# Recent Progress and Advances of Multi-Stimuli-Responsive Dendrimers in Drug Delivery for Cancer Treatment

**DOI:** 10.3390/pharmaceutics11110591

**Published:** 2019-11-08

**Authors:** Ngoc Thuy Trang Le, Thi Nhu Quynh Nguyen, Van Du Cao, Duc Thuan Hoang, Van Cuong Ngo, Thai Thanh Hoang Thi

**Affiliations:** 1 Institute of Research and Development, Duy Tan University, Danang 550000, Vietnam; lenthuytrang4@duytan.edu.vn; 2 Faculty of Pharmacy, Lac Hong University, Buu Long Ward, Bien Hoa City, Dong Nai Province 810000, Vietnam; ds.nhuquynhnguyen@gmail.com (T.N.Q.N.); caovandulhu@gmail.com (V.D.C.); hoangthuand08@gmail.com (D.T.H.); vancuong283@gmail.com (V.C.N.); 3 Biomaterials and Nanotechnology Research Group, Faculty of Applied Sciences, Ton Duc Thang University, Ho Chi Minh City 700000, Vietnam

**Keywords:** smart dendrimer, multi-stimuli-responsiveness, drug delivery, cancer treatment

## Abstract

Despite the fact that nanocarriers as drug delivery systems overcome the limitation of chemotherapy, the leakage of encapsulated drugs during the delivery process to the target site can still cause toxic effects to healthy cells in other tissues and organs in the body. Controlling drug release at the target site, responding to stimuli that originated from internal changes within the body, as well as stimuli manipulated by external sources has recently received significant attention. Owning to the spherical shape and porous structure, dendrimer is utilized as a material for drug delivery. Moreover, the surface region of dendrimer has various moieties facilitating the surface functionalization to develop the desired material. Therefore, multi-stimuli-responsive dendrimers or ‘smart’ dendrimers that respond to more than two stimuli will be an inspired attempt to achieve the site-specific release and reduce as much as possible the side effects of the drug. The aim of this review was to delve much deeper into the recent progress of multi-stimuli-responsive dendrimers in the delivery of anticancer drugs in addition to the major potential challenges.

## 1. Introduction

Cancer has proven to be a significant liability when it comes to causing deaths globally and has risen as a noteworthy general medical issue. Around 14 million new cases were reported in the year 2012 and overall mortality of at least 8.8 million deaths in the year 2015 alone [1,2]. The disease is mainly characterized by irregular and uncontrollable cell development. Furthermore, it is also characterized by threatening phenotypic behavior, for instance, invasion and metastasis. Cancer antagonistically influences various parts of the body with devastating outcomes [3,4]. In a human body, cells have a constrained life expectancy, and recoveries through cell division are essential [1]. With regards to cancer, it is a circumstance whereby cells in a body change and begin to multiply rapidly. These transformed cells group and assemble to form a bump called a tumor [5]. Metastasis, at that point, happens whereby the tumor cells attack different organs in the body and subsequently interfere with substantial capacities that are vital to living. Eventually, the process enables the disease to cause the death of its host [6,7]. Analytical and in-depth research on the treatment of tumors has been carried out for a considerable length of time, for instance, chemotherapy which has been demonstrated to be viable has for several years received a significant amount of interest. However, the main limitation of the current clinical application of chemotherapy drugs is their respective wide bio-distribution and short half-life [8,9,10]. It is therefore critical to develop novel medicine delivery systems to overcome the underlying limitations and also improve the efficiency of the cancer treatment methods that are available.

**Figure 1:**
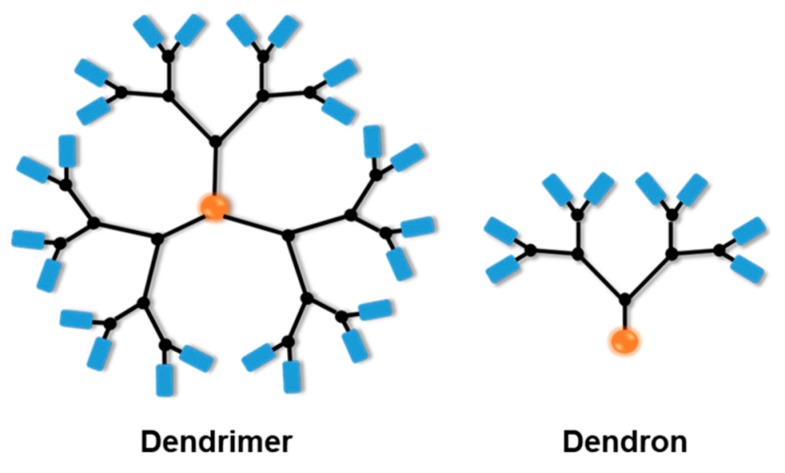
Schematic illustration of the distinct differences between the terms dendrimer and dendron.

Dendrimers have shown a considerably great promise as an efficient carrier with regards to drug delivery as a result of their superior properties as well as their distinctive structures [11]. Dendrimers are repetitively branched molecules (dendrons), but they have the symmetric groups-structure around the core in a spherical fashion (Figure 1). Whereas dendrons are not specific chemical groups nor particular molecules, rather they represent the structure that has multiple terminal groups “branched” out from the core. This core—known as the focal point—is a chemically available group, from which a series of similar functional groups and bonds can be attached. Many terminal groups can derive from the formation of many bonds, linkages with the chemical reactions carried out from the focal point. Dendrons and their structures are often designated to be monodisperse and highly symmetric [12]. With the unique composition, dendrons are subjected specifically to orthogonal reactions serving different purposes. With that diversity in determining terminal groups and flexibility in choosing the chemical reactions, dendrons are subjected to varied preparation processes, especially with stimuli-responsive functional groups as terminal groups to enhance the desired properties that suit different medical purposes. Dendrimers and dendrons have been distinctively engineered as nano-devices, either as drugs or in nano-carrier drug approaches [13,14].

As a drug delivery system, dendrimers’ biological effect is often caused by the terminal moieties and is thus responsible for their universal efficiency. Moreover, to optimize the features and function brought by those moieties, the quality and quantity of the terminal groups are put under strict considerations. Furthermore, it is critical to note that, due to their optimized, reproducible, as well as proper design parameters that tend to overcome the physiological precincts of conventional drugs; for instance, with regards to solubility, therapeutic efficacy, specificity, and bio-distribution, dendrimers are highly successful [15,16,17]. Compared to its close relative, dendrimer possesses certain supremacies over hyperbranched polymers. The polydisperse (different from the monodisperse of dendrimer) hyperbranched polymer although having the same dendritic structure, they are prepared in only one single polymerization step. As a result, hyperbranched polymers experience the imperfect structure of branches and have an imprecise number of terminal groups (rather they are determined by average numbers). Therefore, although having a more expensive cost for producing, dendrimers can employ the properties and functions of the terminal groups than the hyperbranched polymers, thus being better known for their efficiency as a carrier. Conversely, it is also critical to note that unmodified dendrimers as potential drug delivery systems (DDSs) also have some considerable limitations [16]. For example, polyamidoamines or PAMAMs tend to be considered toxic, and at present, this toxicity may only be resolved by modifying the dendrimer configuration [18,19,20]. However, it has long been established that dendritic polymers tend to have enormous potential in several cancer-treating therapies, particularly given their ability to be designed for biological specificity [21]. As such, modified dendrimers lack toxicity, and their unique bio-compatibility makes them have essential qualities for being an effective drug delivery system [22].

Modified dendrimers have proven to be an effective drug delivery system compared to conventional dendrimers. Multi-stimuli-responsive dendrimers’ exclusive structure allows them to efficiently deliver the more therapeutic agents and thus control their release in an efficient manner [23]. In the recent past, an inclusive type of multi-stimuli-responsive dendrimer with pH-, CO_2_- responsiveness was developed [23,24,25]. The development was done through simple modification of polyamidoamine dendrimers (PAMAM) by incorporating various *N*-dialkylaminoethyl carbamate moieties [25,26,27]. PAMAM promise to create a great revolution in the field of medicine since they contain dendrimers characterized by a well-controlled structure. Furthermore, these polymers also have a lower critical temperature based on thermoresponsive. Due to their great structure and unique properties, dendrimers have proven their potential as better drug carriers. Nonetheless, there seems to be a challenge when it comes to accurately control the release of its payload emanating from a dendrimer matrix [8,28,29]. As such, more research ought to be carried out on the topic to determine how this technology can be best applied towards better results.

This review intends to examine the current developments in the use of multi-stimuli-responsive dendrimers as a drug administered for cancer treatment. Furthermore, this review also focuses on some of the available strategies on the synthesis of dendrimers that are capable of controlled drug release at the target site responding to stimuli that originated from internal changes within the body as well as physical stimuli manipulated by external sources. Finally, the potential biomedical applications and promising research of multi-stimuli-responsive dendrimers are also discussed.

## 2. Multi-Stimuli-Responsive Dendrimers in Drug Delivery for Cancer Treatment

Nanosystem responsive to different stimuli is reported to have more effective anticancer effect in targeted therapy than single-stimuli nanosystems. Various studies that have been conducted to evaluate the application of multi-stimuli dendrimers in various processes are summarized in Table 1 [30]. For example, a recent study by Tong et al. investigated the multi-stimuli-responsive dendrimers that contain *N*-dialkylaminoethyl carbamate moieties [25]. The study focused on providing an effective but simple method of developing new multi-stimuli-responsive dendrimers for potential application in smart material.

In 2016, a dual redox and pH-sensitive dendrimer was synthesized by Hu et al. The authors designed the sensitivity of the system by conjugating poly(ethylene glycol) (PEG) to PAMAM dendrimer via redox-responsive di-sulfide linkages. Doxorubicin (DOX) was loaded into the hydrophobic core creating the PAMAM-SS-PEG/DOX complex. In vitro drug release resulted in the release of DOX in reductive environment and low pH. Cytotoxicity test was done on B16 tumor cells, showing a decrease in cellular uptake as PEGylation degree increased. Additionally, in vivo test on mice bearing B16 tumor proved a significant antitumor activity of PAMAM-SS-PEG/DOX [31]. As a similar case described in 2017, Nguyen et al. got initial achievement in the development of heparin-modified PAMAM dendrimer for use in cancer treatment that is responsive to dual stimuli including pH and redox potential [32].

In another article, Hu et al. investigated the DOX-loaded PAMAM-SS-PEG complex. Redox-sensitivity was determined by changes in particle size and zeta potential in reductive environment using dynamic light scattering (DLS). DOX was loaded into the hydrophobic core with loading content of 10% (drug over carrier). In vitro drug release showed significantly higher cumulative DOX released from the conjugates in comparison with the non-cleavable counterparts. Moreover, the DOX-loaded PAMAM-SS-PEG complex demonstrated potential in eradicating B16 and A549 tumors more efficiently. Despite the somewhat less cell uptake, the highest PEGylation degree in PAMAM-SS-PEG conjugates resulted in the best antitumor effect [33].

In a study by Shen et al., dual-sensitive (pH and temperature) poly(β-aminoester) dendrimers were successfully developed. The sensitivities primarily depended on phase changes of the surface groups in response to changes in external temperature or pH. Due to the thermo-sensitivity, hydrophobic drugs could be loaded into dendrimers at low temperature without the use of organic solvents. Drug release from dendrimers occurred slowly and sustainably at 37 °C and physiological pH. However, at lower pH, the drug release process was accelerated [34].

Another study by Wang et al. also evaluated the application of stimuli-responsive dendrimers in the delivery of drugs [35]. The main highlights of this study included the recent advancement and opportunities for developing stimuli-responsive dendrimers for treating several diseases, especially cancer. In addition, Wang et al. provided insight on pH-responsive and super elastic dendrimers that are produced using Cryo-aza-Michael addition reaction method. The authors indicated that the dendrimer cryogels demonstrated stability in acidic pH; however, they degraded relatively quickly in physiological pH. 

Following the results of a recent study, the application of multi-stimuli-responsive dendrimer has been investigated and Fu et al. is the one who evaluated the use of films and hydrogels in drug delivery [36]. The authors indicated that responsive polymers have been designed to disrupt their characteristics upon contact with various biological, physical, or chemical stimuli. Thus, through the incorporation of functional groups, the polymers can sense the prevailing environmental conditions and facilitate the release of encapsulated therapeutic drugs to specific action areas. Fu et al. highlighted various studies that have been conducted to evaluate multi-stimuli-responsive materials, especially hydrogels, films, and particles. 

A similar study by Patil et al. investigated the creation of structurally diverse stimuli-responsive dendrimers using the Passerini multicomponent reaction method [37]. This approach employs the ultraviolet and redox sensitive groups that were integrated into the interior of the dendrimer. The authors used convergent synthesis through a reaction of nonbranched functional molecules and branched repeating units using the azide–alkyne cycloaddition and Passerini multicomponent reactions. In this study, the periphery dendrimer was functionalized using ethylene glycol to produce another dendrimer with hydrophilic peripheral chains and hydrophobic core. In addition, the study also involved photo- and oxidation-triggered degradation of the dendrimer. 

Li et al. also evaluated the application of stimuli-responsive dendrimers for drug delivery in cancer therapy [38]. The authors identified that new DDSs have made significant progress and play a central role in the treatment of cancer. According to Li et al., conventional chemotherapeutic drugs that have several restrictions including short half-life, nonspecific toxicity, and low concentration can be used to effectively transport cancer medication to specific sites in the tumor. Li et al. indicated that stimuli-responsive DDSs can be used to monitor the release of drugs in order to enhance the curative effect, minimize potential damage of organs and tissues, and reduce the side effects of traditional cancer medications. The authors mainly focused on the three categories of nanomaterials based on their structure and physiochemical properties. These categories included multi-stimuli, endogenous stimuli-, and exogenous stimuli-responsive dendrimers [39]. 

Tang et al. reported in a study about the use of stimuli-responsive nanoparticles in drug delivery for cancer therapy [40]. The authors highlighted that stimuli-responsive nanoparticles provide a framework for improving therapeutic efficiency and effectiveness and minimizing the side effects of traditional cancer medication. In this study, Tang et al. summarized the latest advancement in the use of dendrimers in controlled delivery of anti-cancer drugs under different stimuli including redox, pH, light, temperature, among others. 

Finally, Wells et al. also investigated the use of stimuli-responsive dendrimers in drug delivery for cancer therapy [41]. According to Wells et al., stimuli-responsive nanocarriers are designed to release drugs in response to biological, chemical, or physical triggers in the cellular environment. The authors also highlighted current and evolving stimuli-responsive nanomaterials and their potential use in biomedical treatment. 

## 3. Recent Trends of Stimuli-Responsive Dendrimers in Controlling the Release of Drug at Target Sites

**Figure 2:**
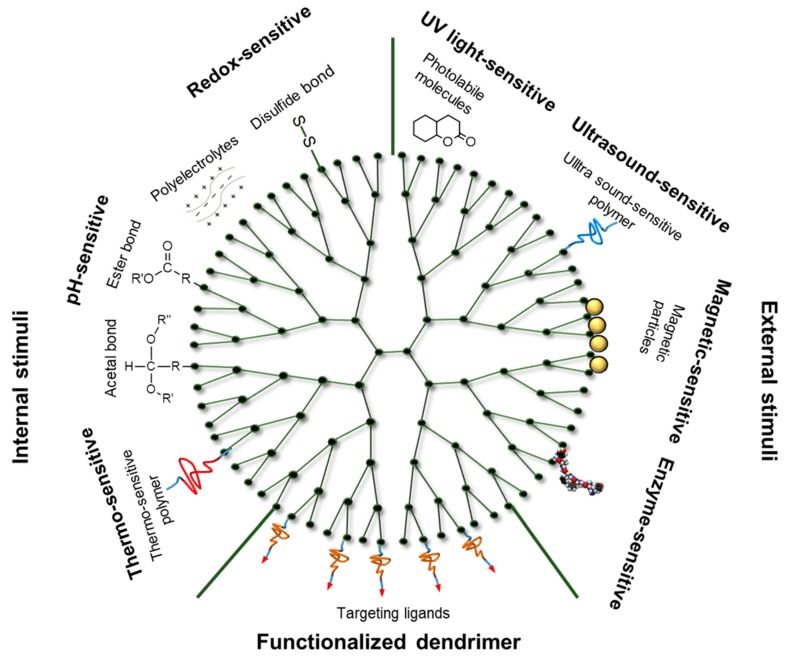
Schematic illustration of stimuli-responsive dendrimer-based drug delivery systems.

Surface functionalization of dendrimers involves the incorporation of many active sites in the dendrimers to form macromolecules that possess multifunctional architecture (Figure 2). Dendrimers that have been functionalized are also called structurally controlled dendrimers and possess more than six nanoscale characteristics known as critical nanoscale design parameters (CNDPs) [42]. These features included rigidity, size, flexibility, architecture, shape, elemental composition, and surface chemistry [43]. The CNDPs can be transformed to produce various new features that are suitable and desirable for a wide range of commercial and industrial processes. Due to the systematic considerations of the process, surface functionalization is normally introduced into the dendrimers’ frameworks at the core or periphery [42]. In addition, the surface functionalization can also be introduced in both the core and periphery [24,44]. Research indicates the essential role played by dendrimer in the formulation of nanoparticles [21]. According to Barman et al., the metal ion is normally incorporated with dendrimer ligands including surface functional groups and interior tertiary amines through processes such as electrostatic or coordination interactions [45].

Various changes occur during surface functionalization. According to Ahmad et al., hydrophilic surfaces allow water to spread evenly because the interaction with the surface becomes stronger than the intermolecular forces in the bulk water [46]. On the other hand, hydrophobic surfaces repel or attract water weakly, producing water beads because the interactions in the surface are much weaker. The processes that occur during surface functionalization include polymer brushes, layer by layer deposition, silane coupling, and anti-microbial surfaces [47]. During coating, hydrophilic materials are deposited to the surface through adhesion or absorption, interpenetration involving the mixing of base polymer and functional material, or mechanical interpenetration. In absorption/adhesion, the functional layer is established physically on the base polymer, while in mechanical interpenetration, pore structures and material layer are added [39,48].

Research indicates that the unique architecture, aqueous solubility, and the numerous chemically versatile surface groups of dendrimers makes the effective delivery agents for therapeutic agents such as anticancer medications [15]. The –NH_2_ groups component of the PAMAM dendrimers guide their interaction with cell membranes that are negatively charged; thus, resulting in permeation of the dendrimers [49,50]. Conversely, the existence of a positive charge on the surface of the dendrimers means that they are cytotoxic to cationic dendrimers; hence, limiting their application in clinical settings. However, research indicates that this limitation can be addressed through surface functionalization or modification [50]. Surface modification in dendrimers involves using dendrimers with functional groups to engineer, disguise the cationic charges, and adapt them into biocompatible dendrimers [24]. This concept has been supported by PEGylation, folate, and peptide conjugation which involve neutralization of charges in the dendrimers surfaces [51,52,53,54,55,56,57]. However, surface functionalization of dendrimers through attachment of molecules is the most common approach [58].

Various studies have been conducted to investigate surface functionalization [59,60,61,62,63,64]. For example, Isabel et al. examines the selective surface functionalization of nanoparticles to improve cell uptake, stability, and pH-responsive drug delivery. Tian et al. also investigated the use of surface functionalized exosomes as drug delivery components for cerebral ischemia therapy [63]. In addition, Fortuni et al. examined how surface functionalization through polymeric engineering of nanoparticles can be applied in efficient drug delivery systems [60]. 

### 3.1. Functionalized Dendrimer with Polymers and Targeting Agents

Surface modification of stimuli-responsive nanosystems with biocompatible polymers and targeting agents is a promising approach to synthesize delivery systems capable of effective delivery, high tumor-targeting selectivity, and low toxicity [65].

In 2012, Chang et al. designed pH-sensitive, tumor targeting PEGylated and folic acid (FA)-functionalized PAMAM dendrimers G3.5. DOX and superparamagnetic Fe_3_O_4_ nanoparticles were co-loaded into the dendrimers. DOX was linked with the dendrimers via hydrazine linkage. This linkage provided ideal pH-sensitivity and could be cleaved under the low pH environment at tumor site. PEG coated on the surface helped improve solubility and stability in liquid phase, hence the enhanced circulation time. Finally, FA as a targeting agent helped in selective binding with folate receptors overexpressed in cancer cells [66]. 

In 2013, a core–shell pH-sensitive FA-PEG-CCTS/PAMAM system with PAMAM dendrimers core coated by FA-functionalized tethered carboxylated chitosan-PEG (PEG-CCTS) shell was successfully synthesized by Wang et al. The core–shell nanostructure was used to deliver HMGB1/pDNA complex in gene delivery application. The results indicated efficient delivery to cancer cells overexpressing folate receptors [67].

pH-sensitive dendrimer functionalized with PEG was also conducted by She W. et al. in 2015 to be applied in DOX targeting delivery to liver cancer tissue. Specifically, DOX was linked to dendrimers via pH-sensitive hydrazine bond, afterwards, PEG was conjugated on dendrimers surface and finally galactose functionalization was performed (Dendrimer-DOX-PEG-Gal). Results showed that the release rate of DOX at pH 5.0 was significantly faster than at pH 7.4 due to the cleavage of hydrazine bonds in response to low pH. The synthesized complex was able to efficiently kill liver cancer cells HepG2 in vitro. In comparison to other dendrimer complexes, dendrimer-DOX-PEG-Gal was functionalized with terminal galactose, facilitating selective drug targeting of HepG2 liver cancer cells, which then improved treatment efficacy and minimized adverse effects [68,69].

In order to improve targeting efficiency to folate receptors on colon cancer cells, oxaliplatin anticancer agent was loaded into G4 dendrimer functionalized with PEG and FA. The synthesized nanosystem had drug loading efficiency of 34% and better drug release in low pH environment. Moreover, cellular uptake test showed good uptaking of the system by colon cancer cells, at 84.67% ± 1.98% [70].

In 2018, PAMAM dendrimer was functionalized with both PEG and targeting agents RGDyC in the study of Huang et al. Results showed that the synthesized dendrimer not only sustained the release of drug but also reduced the cytotoxicity thanks to targeting agent modified on the surface of dendrimer [71].

In a study of Wang et al., Pluronic P123 was used to modify PAMAM dendrimer. The system can self-assemble and encapsulate DNA, forming a complex in nano size (100–250 nm). Cytotoxicity and DNA transport efficiency were evaluated and compared to PEI and PAMAM dendrimer. Results showed cytotoxicity to cancer cell lines including MCF-7, HepG2, and 293RT with every complex. In addition, PAMAM functionalized with a small amount of P123 could significantly improve DNA delivery efficiency in comparison to non-functionalized PAMAM [72].

In 2016, Wang et al. designed a nanocarrier system from PAMAM dendrimer functionalized with Pluronic F68 for DOX-delivery against multidrug resistant cancer. DOX activity after encapsulation was evaluated upon the MCF-7/ADR multidrug resistant cancer cell line. Results indicated higher accumulation of DOX delivered by the carrier system at tumor site and superior anti-tumor ability than free DOX [73].

Throughout many years of research, there are many nanosystems that are capable of controlled release to destroy cancer cells and reduce side effects; however, the optimization to develop a nanosystem responsive to different stimuli based on PAMAM still attracts much attention of many research groups. Among them, research on the multi-stimuli-responsive materials, especially conjugating thermo-sensitive polymers (Pluronic or NiPAM) to the surface of PAMAM via redox-sensitive, pH-sensitive, or UV-sensitive linkages still has not been studied yet. 

Dendrimers respond to various stimuli signals that can be categorized as either external or internal (Table 2). The significant differences in the intracellular environment between normal and tumor tissues cause variations in the redox state, pH, and the amount of biomolecules. External triggers include ultrasound, magnetic fields, and photo triggers. Internal triggers may include enzymes, redox, and pH.

### 3.2. Internal-Stimuli-Responsive Dendrimers

#### 3.2.1. Redox-Sensitive Dendrimers

**Figure 3:**
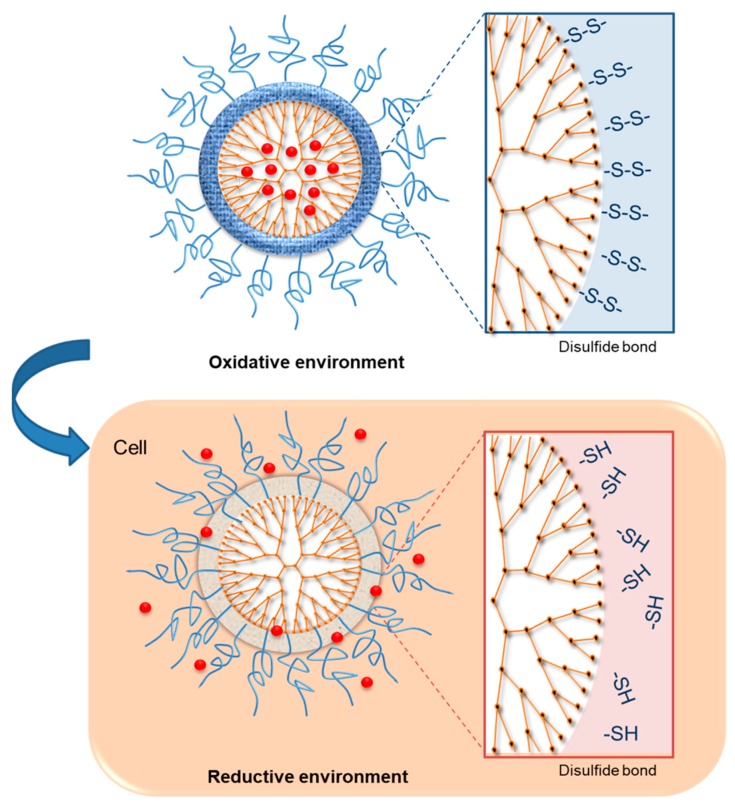
Dendrimers capable of redox-dependent drug release [105].

The difference in the concentration of glutathione (GSH) inside and outside of the cells is utilized to control the intracellular delivery of drug [32]. Since the intracellular compartments contain a high concentration of GSH (about 2–10 mM), which is 100–1000 times higher than extracellular environment and bloodstream (about 2–20 μM), GSH is considered as an ideal stimulus to quickly destabilize the redox-sensitive nanogel inside of the cells, promoting the effective intracellular release of the drug [98,99]. Reduction-sensitive dendrimers and conjugates have been identified as a fascinating group of biomedical materials that can be applied in intracellular triggered drug and gene delivery [51]. The rationale for the design of reduction-sensitive dendrimers involves the integration of disulfide linkages in the cross-linker, main chain, or side chain (Figure 3) [100]. Redox-sensitive dendrimers are mainly characterized by exceptional stability in extracellular fluids and in circulation [101]. However, these dendrimers are susceptible to rapid degradation in reductive environments inside the cell such as the nucleus and cytoplasm [102]. The reaction between the negatively charged cell-membrane and positively charged dendrimers results in membrane disruption, cell lysis, and hemolytic toxicity, which are the disadvantages of the PAMAM dendrimer DDS [49]. Some limitations of the DDS that need to be addressed include undesirable drug release, high toxicity, and unsatisfied drug loading. To minimize toxicity, PAMAM dendrimers are usually modified with substances such as fluronic, PEG, and heparin, by preventing contact with protonated amine groups located in the cell membrane; thus, enhancing biocompatibility [49,103,104].

In 2013, Wang X. et al. synthesized a redox-sensitive PAMAM dendrimer encapsulating gold nanoparticles (DEGNPs). The system was used to deliver anticancer agents with thiol groups, such as captopril and 6-mercaptopurine (6-MP) via Au–S bond. The encapsulated drug established an “OFF-ON” pattern during release in the presence of reductive agent. Results of flow cytometry confirmed the system’s capability of drug release intracellularly, where GSH concentration is much higher than extracellular environment [74]. 

Yan et al. reported a self-assembled nano dendrimer system containing hydrophobic di-sulfide units and hydrophilic polyphosphate units. The synthesized dendrimer had a size range of 30–78 nm and a very narrow size distribution. In addition, the resulting system was able to assemble and disassemble in a highly reductive environment. The encapsulated hydrophobic drugs were quickly transported into cancer cells’ nuclei with high efficiency, while intracellular GSH triggered drug release [75]. 

In 2015, Wang et al. made use of disulfide linkage to conjugate branched poly(ethylene glycol) with G2 dendrimers. The synthesized system established complexation with nucleic acid and high encapsulation efficiency. The cleavage of the redox-sensitive disulfide linkage enabled the release of drugs and nucleic acid into the intracellular environment, which has a high concentration of GSH. The PEGylation of dendrimer promoted the stability and residence time, compared to native Gs dendrimer. More importantly, co-delivery of DOX and B-cell lymphoma 2 (Bcl-2) siRNA displayed synergistic effect, destroying murine tumor more efficiently than individual DOX or siRNA treatment [76]. 

In 2018, Du et al. developed redox-sensitive dendrimers for delivery of siRNA. The cationic hydrophilic head of Janus dendrimer (ssJD) creates a strong and stable conjugation with anionic siRNA thanks to electrostatic interaction. Loaded siRNA was released from the dendrimer in the redox condition inside tumor cells due to disulfide linkages [106].

#### 3.2.2. pH-Sensitive Dendrimers

The pH of cells plays a major role in the efficient functioning of body tissues [107]. The pH of normal tissues is significantly different from that of diseased tissues resulting from infection, cancer, or inflammation. The intracellular and extracellular pH can be interfered with because of certain diseases [107]. The pH difference in the normal and diseases cells facilitates the delivery of conjugated or encapsulated drugs without disrupting other normal cells [51]. It is known that the endosomal and lysosomal components, and the micro-environments in tumors have acidic pH [107]. As a result, drug delivery vehicles that may be triggered by pH variations are effective and highly desired for treating tumors [107]. Dendrimers are formed using various functional groups, whose pH-dependent release is achieved by disturbing the amphiphilicity of the environment [108]. The disruption of the amphiphilicity of the system can be achieved through the cleavage of primary covalent bonds or interfering with the charges in the dendrimer [109]. When dendrimers are placed in acidic pH, the protonation of tertiary and secondary amines causes the dendrimer to be more hydrophilic. As a result, the changes facilitate the releasing of encapsulated pyrene [21].

Amine-terminated PAMAM dendrimers are pH-sensitive because of the large amounts of peripheral primary amines and internal tertiary amines [49]. PAMAM dendrimers and PEGylated counterparts are used in solubilizing hydrophobic drugs in a pH-dependent way including methotrexate, 2-naphthol, nitronic acid, and nifedipine [110,111]. The reason behind the pH-dependent solubilization is that hydrophobic drugs can be encapsulated effectively into the internal cavities of the PAMAM dendrimers even at high pH [49,107]. In addition, the drugs can be released because of the open structures resulting from the repulsion of protonated amine groups. This reasoning has also been applied to design pH-sensitive PAMAM dendrimers that can sustain additional stable drug encapsulates in neutral pH compared to acidic pH [49].

PAMAM dendrimers are composed of many peripheral reactive groups that can be functionalized using specific ligands and used for tumor cell targeting, prolonged residence in blood circulation, modulation of cellular interaction, and spatially controlled drug release [112]. The groups are used in the conjugation of anti-cancer into PAMAM dendrimers via pH-responsive bonds [49]. This process occurs because a large number of anticancer drugs can be bonded [51,113]. Researchers have evaluated various acid-labile linkers during the development of PAMAM-bound anticancer drug conjugates such as ester, imines, oximes, hydrazones, and cis-aconitic anhydride. The presence of pH-sensitive bonds in between PAMAM dendrimers and anticancer drugs maintains the conjugated drug in the fluids outside the cells while facilitating drug release in lysosomes or endosomes to the tumor cells after internalization [51].

**Figure 4:**
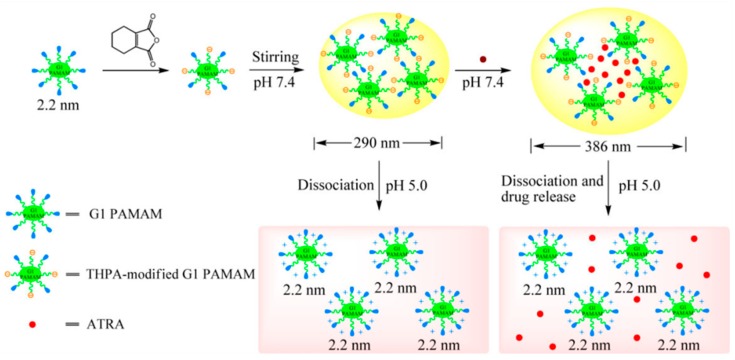
Assembly of 3,4,5,6-tetrahydrophthalic anhydride-modified G1 polyamidoamine dendrimer and pH-sensitive release of drugs, reproduced with permission from [77]. 2019, John Wiley and Sons.

In 2013, a pH-sensitive PAMAM dendrimers was synthesized by conjugating 3,4,5,6-tetrahydrophthalic anhydride (THPA) to the first generation PAMAM dendrimer (Figure 4). Post-functionalized dendrimer was capable of self-assembly to form spherical structure of 125–435 nm in size. Although the synthesized system was considerably stable in physiological pH (7.4), degradation was observed in acidic environment (pH 5.0–6.0) [77].

Contributed to the same purpose, the pH-sensitive PAMAM dendrimer coated iron oxide nanoparticles were developed by the group of Pourjavadi et al. in 2014 by covalently conjugating of DOX to dendrimer via hydrazine bonds. This system has high drug loading capacity and controlled release [78].

In 2015, She W. et al. developed a dendrimer having pH-sensitive property from hydrazine linkages. Results showed that the release rate of loaded DOX in the condition of pH 5.0 was significantly faster compared to that at the condition of pH 7.4 due to the cleavage of hydrazine at low pH [68].

In 2016, Qi et al. developed a carboxymethyl chitosan-modified dendrimer in order to alter the surface charge of dendrimer from positive to negative, thus improve the compatibility of the system. The designed dendrimer had a tendency of accumulating at the tumor site thanks to the enhanced permeation and retention effect (EPR) within cancer tissue. Additionally, the low pH environment at tumor site induced the removal of the carboxylmethyl chitosan coating. The surface charge of dendrimer regained a positive charge, facilitating high intracellular uptake in tumor cells [114].

Similarly, Liu et al. lowered the surface charge of amine-terminated PAMAM dendrimers using zwitterionic chitosan (ZWC). ZWC is a pH-sensitive polymer, hence the dissociation of the ZWC–PAMAM complex upon the low pH environment at tumor site, which allowed PAMAM dendrimers to efficiently enter cells and improved its therapeutic efficacy [115].

#### 3.2.3. Thermo-Sensitive Dendrimers

The state of thermo-sensitive nanocarriers in modern DDS is not only based on the ability as carriers but also in improving sustained release of drugs, circulation time, and passive target-specific delivery [116]. The introduction of thermo-sensitive dendrimers in the preparation of thermo-sensitive nanocarrier systems has improved the ability of cells to respond to changes in temperature in the external environment [117,118]. Thermo-sensitive dendrimers can respond to changes in temperature in the extracellular environment, resulting in significant alterations in its features including conformation, hydrophilic or hydrophobic balance, and solubility [15]. These properties are quantitative in general and are explained using the lower critical solution temperature (LCST) [117]. Thermo-sensitive dendrimers and polymers may exhibit the LCST phenomenon, above which the dendrimer solution is separated in phases due to the aggregation and collapse of the dendrimer chains and expunged water [117].

Thermosensitive dendrimers undergo various solubility changes elicited by variations in temperature [15]. These temperature-dependent changes in solubility involve highly attractive molecules that are used to develop effective thermo-responsive materials [119]. Thermo-sensitive dendrimers could be used in biomedical processes including programmed drug delivery due to the influence of thermotherapy on the temperature of the site of action [120]. Researchers have developed various polymers that undergo transition under fluctuating temperatures such as poly (NiPAM) [79]. For instance, Dwivedi and Singh conducted a study to develop a temperature-sensitive dendrimer by using poly (NiPAM) chains to decorate the dendrimers’ amine end groups [80]. Thermo-sensitive dendrimers have been shown to possess the ability to encapsulate guest polymers, whose catalytic behavior is influenced by temperature changes. According to Karimi et al., thermo-sensitive dendrimers that have narrow transition temperatures can be applied in sensing and drug delivery [121].

**Figure 5:**
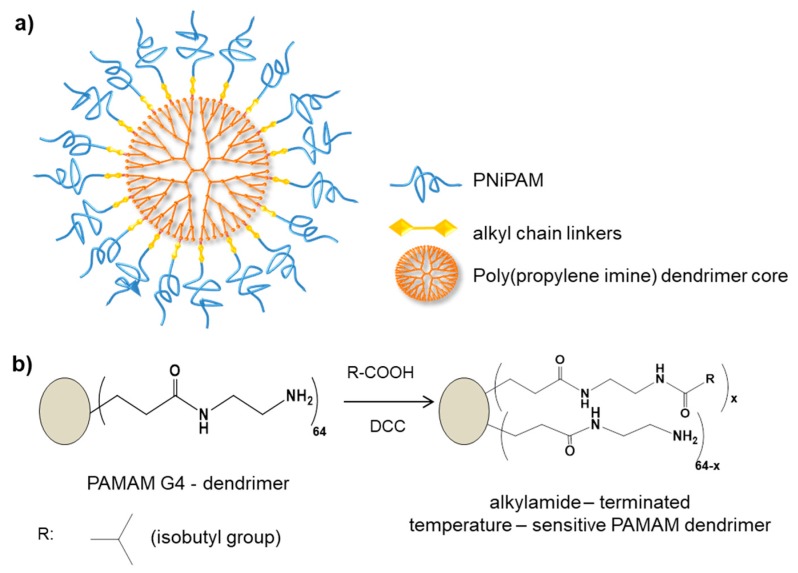
(**a**) Chemical structure of poly(propylene imine) (PPI) dendrimer decorated with temperature sensitive poly(NiPAM) groups and (**b**) schematic representation of a generation 4 (G4) PAMAM dendrimer decorated with a temperature-sensitive isobutyramide (IBAM) groups [122].

Rajasekhar et al. developed self-assembled amphiphilic dendrimers to form thermo-sensitive nanostructures. The synthesized dendrimers contained hydrophilic PEG chains and hydrophobic decyl chains on every repetitive unit of dendrimers (Figure 5). The thermo-sensitivity of the synthesized dendrimers was shown to rely on the generation of dendrimer. Results showed that the higher the generation, the lower the LCST [122].

In another study, Kono et al. reported thermo-sensitive PAMAM and Poly(propylene imine) (PPI) dendrimer coated with isobutyramide (IBAM) groups. The study demonstrated that the thermo-sensitivity of these dendrimers not only depended on temperature, but also directly developed on the generation of dendrimers and environmental pH, since these factors affected the hydrophobic–hydrophilic equilibrium directly [82].

Zhao et al. utilized two thermo-sensitive groups, 4-(isopropylamino)-4-oxobutanoic acid (IPAOBA) and 4-(diethylamino)-4-oxobutanoic acid (DEAOBA) to improve the thermo-sensitivity of the PAMAM dendrimer G2 and G3. Due to a lack of surface groups, PAMAM dendrimers were pre-functionalized with hydroxymethyl aminomethane to increase the number of hydroxyl groups on the surface of dendrimers. The two thermo-sensitive groups above were then conjugated with the hydroxyl terminals of dendrimers [83]. 

Recently, Parham et al. prepared thermo-sensitive high generation dendrimer for use in extraction of rivaroxaban from human fluid and pharmaceutical samples. In this study, polyNIPAM acts as thermo-sensitive agent was utilized to conjugate onto PAMAM, resulting in the thermo-sensitive property of the synthesized system [81].

Researchers have found that thermosensitive dendrimers are useful in drug delivery because of beneficial properties including low toxicity, prevention of overdoses, and being specific to one target. Dendrimers with LCST can be used to trigger smart nanoparticles, which, in turn, can be simulated using temperature [121]. In addition, thermosensitive dendrimers can be employed in delivering drugs to cancerous cells and to enhance the cytotoxicity.

### 3.3. External-Stimuli-Responsive Dendrimers

#### 3.3.1. UV Light-Sensitive Dendrimers

Light can act as an external source of change when designing stimuli-sensitive materials because it is non-invasive and can be controlled remotely [123]. In addition to UV, other light triggers include near and visible infrared depending on their wavelengths [35]. An example of dendrimer conjugate that is sensitive to UV light is ortho-nitrobenzyl (ONB), which is mainly utilized in the manufacture of light-responsive materials [51,84]. The dendrimer is quickly cleaved by UV lights between the wavelengths of 254 and 365 nm [35]. There is also a possibility of conjugating anti-cancer medications such as doxorubicin to dendrimers using ONB linkers. Lin et al., stated that the conjugates exhibit insignificant toxicity in cells when placed in the dark. After UV light irradiation, the ONB linkage’s cleavage between doxorubicin and the dendrimer is followed by the release of the drug [123]. 

In the study by Li et al., PAMAM dendrimer of the generation G3, G4, and G5 were synthesized and peripheral modified with photo-cleavable o-nitrobenzyl (NB) via the reaction between o-nitrobenzaldehyde with amine groups of PAMAM. These photo-cleavable NB were successfully conjugated onto the surface of dendrimer with the grafting efficiency of approximately 100%, forming a photo-responsive shell outside of PAMAM. This shell could be cleaved under the UV treatment at 365 nm. The system was used to load both salicylic acid (SA) and adriamycin (ADR). Results showed that the loading efficiency for the guest molecules including SA and ADR of PAMAM G4.0 was the highest in comparison to the rest. Moreover, results also revealed that the release rate of PAMAM G4.0 for SA was higher in the UV-treated samples compared to non UV-treated samples [85].

Yesilyurt et al. designed photo-responsive dendrimer containing hydrophilic PEG chain and hydrophobic alkyl chain, in which the hydrophobic chain will be linked with backbone of dendrimer via photo-cleavable ortho-nitrobenzyl group. This dendrimer system is capable of aggregating into micelles in aqueous medium and incorporating model drug Nile red inside. Upon the UV irradiation at wavelength of 365 nm, the cleavage of photo-cleavable group in the linkage between dendrimer backbone and hydrophobic chains will destroy the hydrophilic–hydrophobic balance of dendron, thus destroying the micelles structure and releasing the loaded drug. When testing the release profile, results showed that there is no release of drug in response to UV light for dendrimer that do not contain photo-cleavable groups. This proved that the cleavage of o-nitrobenzyl group will help to release the guest molecules from dendrimer upon the UV irradiation [86].

Gao F. et al. (2018) synthesized UV light-responsive PAMAM dendrimer for tissue adhesive by conjugating 4-(3-(trifluoromethyl)-3H-diazirin-3-yl) benzyl bromide onto amine groups on the peripheral of dendrimer (PAMAM-g-diazirine) [87]. 

As reported in one of the studies, Kimet et al. developed dendrimers functionalized with photo-responsive groups including aso-nitrobenzyl and diazobenzene at the focal point. These dendrimers can undergo the morphological transformation from vesicle to fibrillar structure upon the irradiation. This property of dendrimers can control the release of guest molecules encapsulated inside the dendrimer [88].

Some dendrimers also possess a photochemical internalization effect. According to Gonzalo et al., dendrimers that are composed of a porphyrin core are usually restricted in the endosome’s membrane after entering the cell [124]. Upon contact with UV irradiation, the dendrimers may produce ROS, a component that is responsive to facilitate membrane disruption [51]. This characteristic can be applied in cytoplasmic drug and gene delivery through a photochemical internalization effect [124]. 

#### 3.3.2. Ultrasound-Sensitive Dendrimers

Ultrasound provides a few benefits for drug delivery through thermal induction, gas vaporization, and mechanical stimulus [125]. These three processes can also be used as adjunctive stimuli during drug delivery [89]. Due to the ability to combine mechanisms, ultrasound facilitates improved permeation of tissues, specialized barriers, and cell membranes including blood drain barriers [124]. In some instances, ultrasound can activate medications such as haematoporphyrin and 5-aminolevulinic acid, and can also be applied in guided therapy [89]. In 2015, Huang B. et al. developed a novel transdermal gel for the delivery of diclofenac comprising a PAMAM dendrimer. The DF gel was coupled with sonophoresis to enhance the permeation of DF through the skin. Hairless male Wistar rat skin was used to study the transdermal permeation of DF. The results indicated that after 24 h, about 257.3 µg/cm^2^ cumulative drug permeation was observed with DF-dendrimer gel without ultrasound treatment, while a significantly higher amount (935.21 µg/cm^2^) was observed with rat skin models treated by sonophoresis [90]. 

In 2017, Manikkath et al. successfully combined PAMAM dendrimers and low-frequency ultrasound on transdermal delivery of ketoprofen. Both PAMAM dendrimers and sonophoresis could individually improve the drug transdermal permeation, but the permeation was dramatically higher when combining dendrimers and ultrasound treatment [91].

Recent evidence indicates that the use of ultrasound in cancer therapy is advantageous in chemotherapeutic drug delivery and facilitates positive outcomes including complete eradication of tumors and decrease of tumor growth [92,93]. In addition, ultrasound has been applied in facilitating healthy tissue regeneration; for example, synergistic therapy [94].

#### 3.3.3. Magnetic-Sensitive Dendrimers

Magnetic nanoparticles are a common group of nanomaterials, popular for their biocompatibility and high magnetization, which has made them applicable in various biomedical processes including immunoassay, drug delivery, MRI, hyperthermia, cell separation, biosensing, and detoxification of biological fluids [126]. Conversely, dendrimers are created from a core containing repetitive branching units known as monomers, which forms a globular structure [127]. Due to their controlled size and structural features, dendrimers have become a suitable elements for biomedical applications especially for carrying therapeutic materials [92,126]. Researchers have been trying to combine the features of magnetic nanoparticles and the unique structure of dendrimers to develop a facile platform for improved biomedical applications and therapeutics [127]. In a study by Li et al. in 2018, PAMAM dendrimer coated functionalized iron oxide Fe_3_O_4_@SiO_2_-NH_2_ nanoparticles were synthesized via Michael addition and amidation. Enzyme penicillin G acylase was immobilzed on the carrier by cross-linking agent glutaraldehyde [95]. 

Targeted drug-delivery systems have the potential to effectively transport drugs to their desired locations, improve patient adherence, minimize healthcare costs, and possibly extend the product’s life cycle [128,129]. However, drug delivery using dendrimers has stagnated because the majority of drugs being used have extremely low solubility, high toxicity, and are excreted rapidly [15]. Current drugs are also inhibited by other factors including untargeted biodistribution, in vivo degradation, non-specific drug delivery, and the short circulations of half-lives. Glassman et al. indicated that these limitations could potentially be addressed by introducing PEGylation in the polymer systems, which has been found to increase targeting, solubility, and pharmacokinetics of drugs [130]. 

#### 3.3.4. Enzyme-Sensitive Dendrimers

Enzymes play an important role in all biological processes [30]. Theocharis et al. indicates that the up-regulation of enzyme expression is linked with various illnesses; for example, various tumors contain an over-expressed amount of cathepsin B and matrix metalloproteinases (MMP). Therefore, enzymes can be used as triggers when designing stimuli-responsive dendrimers. It is possible to conjugate medications like DOX to dendrimers through peptide linkers such as collagen, facilitating the delivery of drugs to tumors to destroy cancer cells and prevent tumor growth. 

In 2014, Zhang et al. synthesized mPEGylated peptide-functionalized dendrimers. DOX was conjugated to the periphery of dendrimer via an enzyme-sensitive linker Gly-Phe-Leu-Gly (GFLG). The mPEGylated dendrimer-DOX complex was able to self-assemble forming nanoparticles [96]. 

In 2017, Zhang et al. continued developing enzyme-responsive lysine-functionalized and PEGylated dendrimers conjugated with gemcitabine (GEM) via an enzyme-sensitive linkage. The PEGylated dendrimer-GEM complex showed more rapid drug release in tumor cellular environment, which secreted Cathepsin B capable of cutting enzyme-sensitive linkage. This led to 80% higher in GEM released in Cathepsin B contained environment in comparison to non-Cathepsin B environment after 24 h. The dendrimers were also able to enhance anti-tumor effect when incubated with 4T1 breast cancer cells on mice [97].

Normally, bacteria in the human body produce hydrolytic or reductive enzymes that can break down different types of polysaccharides including dextrin, chitosan, and pectin [131,132]. An example of reductive enzyme is azo-reductase, while hydrolytic enzymes may include glycosidases [8,92]. In most enzyme-sensitive polymer systems, it is the enzyme itself that is used to break down polymers or their assemblies [30]. The primary benefits of enzyme-responsive polymers are that their decomposition is not dependent on external triggers, they possess high selectivity, and can be used in mild conditions.

## 4. Current Advances in General Biomedical Applications of Multi-Stimuli-Responsive Dendrimers

Dendrimers can be conjugated with several chemical species including detection and imaging agents, biomolecules, therapeutic agents, targeting components, and pharmaceutical agents [30]. The structure of dendrimers is three-dimensional, a characteristic that gives them various unique properties including nanoscaled globular shapes, hydrophilic–hydrophobic cavities on the interior, well-defined peripheral functional groups, and exceedingly low polydispersity [127]. These features allow dendrimers to be used in various applications. 

**Figure 6:**
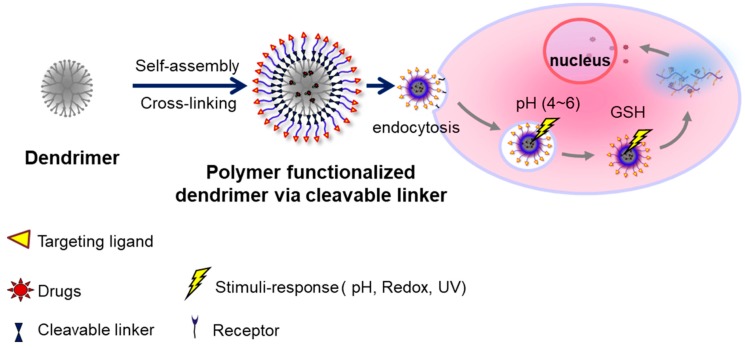
Illustrated scheme of dendrimer responsive to multi-stimuli such as pH, redox potential, ultraviolet (UV), and capable of tumor-targeting and controlled release of drug.

There are various potential applications of dendrimer technology in biomedical and industrial fields. The most promising for utilization are the PAMAM dendrimers that are used to transport drugs to target tissues [133]. Dendrimers have been used in various pharmaceutical applications; for example, antitumor/anticancer medications and nonsteroidal anti-inflammatory drugs (NSAIDs) [134]. The use of dendrimers as drug carriers enhances the pharmacokinetic features of drug particles; thus, decreasing potential side effects through surface modification using different ligands. This process enables drugs to target specific tissues and tumor/cancer cells. Dendrimers may also be used as tools for delivering contrast agents and genetic materials during magnetic resonance imaging (MRI) [113]. Overall, dendrimers play a central role in drug delivery, tissue regeneration, and diagnostics (Figure 6).

### 4.1. Drug Delivery

Dendrimers are nanoparticles and have numerous benefits over microparticles and other elements because of their small size and relatively easy cellular uptake through endocytosis [133]. This process allows the transportation of drugs into the cells [135]. Dendrimers are branched macromolecules with a central core unit which has a high molecular uniformity, specific shape and size features, distribution, a highly- functionalized, terminal surface, and narrow molecular weight [133]. The development of dendrimers has facilitated effective control of the distribution of drugs which has been beneficial in eliminating the limitations of traditional medications. Dendrimers, unlike traditional polymers, have been widely applied in biological processes because of their biocompatibility, high water solubility, precise molecular weight, and polyvalency [135]. These characteristics make dendrimers ideal carriers during drug delivery and targeting processes [12,21,92,136]. When evaluating the use of dendrimers in drug delivery, their biopermeability in various biological membranes should be considered. 

Various factors make dendrimers the most suitable carriers in drug delivery. According to Prajapati et al., dendrimers have well-defined nanostructures and control over their sizes, surface functionality, and branching density. Dendrimers are extremely beneficial nanoscale carriers during gene and drug delivery into cells. Dendrimers can be formulated with both phrophobic and hydrophilic and drug molecules. 

Dendrimers can be used in various drug delivery applications including oral, intravenous, nasal, transdermal, pulmonary, and ocular drug delivery systems. The huge potential of dendrimers as nanocarrier systems is because of their ability to cross cell barriers through both transcellular and paracellular pathways. In addition, statistical modification and optimization can be performed on the ratio and number of dendrimer surface groups that affect controlled release, biodistribution, and receptor mediated targeting of drugs from the interior of the dendrimer [135].

The three-dimensional nature of dendrimers provides them with various unique properties for instance globular features that can be modified by different dendrimer generations. This characteristic provides dendrimers similar shapes and sizes with specific biomolecules and proteins; thus, giving them similar perfection to biomimics [137]. In addition, the tendency of dendrimers to have branching patterns infuses these features with various periphery functional groups, allowing for the delivery of drug molecules [138].

Dendrimers can be used to encapsulate drugs for delivery into specific tissues in the human body. Alternatively, dendrimers can be utilized as time-release vehicles for agents that are active biologically. For example, despite having adverse side effects, the 5-Fluorouracil (5FU) dendrimer has a significant anti-tumor activity [139]. After acetylation, PAMAM dendrimers can be transformed into dendrimer-5FU conjugates and used for drug delivery (Noriega-Luna et al. 50). Due to the solubility of dendrimers, hydrolysis of the conjugates will release 5FU, which can be useful for carrying anti-cancer drugs.

Dendrimers that are used to deliver drugs to target tissues have various advantages. First, drug delivery dendrimers can attain moderately uniform distribution of doses of drugs in their targets. Second, dendrimers can also achieve high solubility of the target drug compared to their own aqueous solubility. Third, dendrimers can facilitate sustained drug release which minimizes the dosing frequency. Fourth, dendrimers are well suited for delivering macromolecules and have minimal side effects. Fifth, dendrimers have been found to improve patient compliance and facilitate drug internalization by cells.

### 4.2. Diagnostics

Martinho et al. highlighted that dendrimers possess a 3D branched topology that has unique chemical and physical properties, multiple functionalities, and globular shape that makes them useful as catalysis, materials, and in biological processes. Over the past few decades, there has been an increasing focus on the application of dendrimers in diagnostics including magnetic resonance imaging (MRI), X-ray, and radiotherapy [113]. Dendrimers have been widely applied to carry various molecular pharmaceuticals which have various benefits in therapy and diagnosis. For instance, the interior cavities and amphiphilic property of dendrimers can be applied to encapsulate hydrophilic or hydrophobic drugs depending on the specific component. Additionally, given that dendrimers are monomolecular, they can evade drug formulations instability using traditional amphiphilic polymers. Conversely, dendrimers possess linear correlation that is suitable for some biomedical applications such as MRI [113]. According to Noriega-Luna et al., medium sized dendrimers can be used for MRI contrast agents during the diagnosis of lymphatic systems. Moreover, dendrimers are highly branched and multivalent, making them suitable for tissue regeneration and as cross-linking agents.

Dendrimers have previously been used to transport small molecule pharmaceuticals to target tissues. These molecules are beneficial as scaffolds or carriers in therapy and diagnosis. A good example is the utilization of dendrimers’ interior cavities and amphiphilic characteristics to encapsulate hydrophilic and hydrophobic drugs. Additionally, the monomolecular polymer micelles characteristic of dendrimers allows them to avoid the instability resulting from drug formulations by utilizing traditional amphiphilic polymers. Conversely, the linear correlation among dendrimers implies that they can be employed for specific applications. A good example is the application of medium-sized dendrimers during MRI for lymphatic system diagnostics. 

Recently, scientists have combined imaging science and polymer chemistry to construct polymer-based bio-imaging probes that are used for the diagnosis and treatment of various health conditions [134]. The main goal of in vivo imaging is to attain reliable and highly sensitive imaging strategies that are effective in diagnosis in personalized drugs for drug delivery after their dispersion and monitoring therapy [134]. As a result, there is need for further research in the development of dendrimers with the appropriate stealth to avoid opsonization before they reach their intended targets.

### 4.3. Tissue Regeneration 

Dendrimers have also been applied in tissue regeneration through tissue engineering [140]. Although dendrimers have been used widely in biomedical applications, their use in tissue regeneration through scaffolds has been limited [141]. The property that allows dendrimers to be used in tissue engineering is their highly branched and multivalent nature. This property makes dendrimers suitable for various tissue engineering applications such as surface chemistry, crosslinking agents, and as modulators of surface charge. In addition, dendrimers can be used as the main components in tissue engineering scaffolds that mimic the properties of natural extracellular matrices. Similar to linear polymers, dendrimers can also provide doctors with more control over factors including biodegradation profiles and proliferation rates by systematically varying their end group chemistry, concentration, and generation size [142]. The integration of dendrimers into traditional scaffold polymers such as carbohydrates, synthetic polymers, and proteins has resulted in the creation of hybrid scaffolds that possess new biochemical, physical, and mechanical features [140]. 

An example of the application of dendrimers in the biomedical field is the development of anticancer drugs [1]. In the treatment of cancer, dendrimers are used to deliver drugs to specific action sites [143]. A major problem in the current healthcare system is the development of modern medicine that can enhance the pharmacokinetic properties of cancer drugs [1]. As indicated by Choi, Young, and Hyo-Kyung, drugs that are conjugated with dendrimers have prolonged half-life, improved solubility, decreased antigenicity, low immunogenicity, and higher stability [144]. Due to the increased permeability of tumors resulting from its pathphysiological characteristics and hypervascularization, passive targeting of cancer drugs can be performed effectively with the help of dendrimers [145]. As presented by Araújo, Renan et al., the conjugated dendrimers demonstrate high solubility which allow drug deliver and increase the chances of inhibiting tumor growth [15].

Natural and synthetic polymeric scaffold compositions can be applied in tissue engineering. Natural scaffolds are composed of glycoproteins, carbohydrates, or general proteins [146]. The most commonly used protein in the development of scaffold is collagen. However, the use of fibrin is also common because of its ability to transform into mesh-like networks [134]. Chondroitin sulfate and hyaluronic acid are two structural components that have been widely applied in tissue regeneration. The main goal of tissue engineering is to ensure that encapsulated cells can regenerate native ECM, which would eventually replace the scaffold. Thus, the scaffolds must biodegrade at a rate consistent with the biosynthesis of the new ECM.

## 5. Conclusion and Perspectives

This review has demonstrated the application of various stimuli-responsive dendrimers in drug delivery, therapy, diagnosis, and tissue engineering. However, these applications are still on the infancy and conceptualization stages. Therefore, further comprehensive studies should be conducted to demonstrate the therapeutic efficiency of multi-stimuli dendrimers and how their application should be conducted. Future studies should focus on standardized synthesis of PAMAM-based conjugates and addressing toxicity issues. There is also need for increasing the pace of commercialization of dendrimer-based products which has lagged mainly cause of high cost of production. 

Despite dendrimers being investigated for their role in drug delivery systems in the treatment of tumors and cancer, there is significantly fewer studies in the treatment of parasitic infections. For example, multi-stimuli sensitive dendrimers can be applied to minimize toxicity in the treatment of parasitic infections such as toxoplamosis, caused by amphotericin B [147]. The dendrimers’ ability to select specific delivery sites can be optimized to ensure effective transfer of drugs to the phagocyte’s reservoir cells [123]. Future development of vaccine carriers could also increase the use of dendrimers to improve their effectiveness in preventing infections such as schistosomiasis. 

According to the results from a study by Madaan et al., the opportunities for future application of dendrimers in nanomedicine is highly dependent on the productivity of the final component features [148]. Thus, it is essential to design well-defined dendrimers that are multifunctional in order to have the opportunity to advance the use of dendrimer-based products into clinical trials. These dendrimers should also possess a controlled location and amount of drugs, imaging agents, and targeting groups among other important themes. Recent advancements in the production of synthetic tools continue to defy the traditional notion that the synthesis of dendrimers is expensive, slow, tedious, and complex. Today, a very small amount of dendrimers that are used in therapy are cost-effective, providing a promising cost-benefit relationship, and further advancement of the products. However, the future synthesis of dendrimers is expected to be both exciting and challenging because of their effectiveness and the high expectations by stakeholders.

Previous research evidence regarding the application of dendrimers in biomedicine indicates the potential for development of newer and more advanced generation polymers and elicits optimism for future use of these polymers in diagnosis, therapy, and drug delivery. Various studies addressed the application of dendrimers in various delivery methods including ophthalmic, transdermal, oral, and gene delivery. However, there is need for close and more constant attention to drug delivery. Though dendrimers have proven beneficial in previous applications, their use in clinical settings has not attained the success of liner polymers. Thus, there is need for further research on the biomedical and industrial application of dendrimers. With expert supervision and improved synthesis, dendrimers can potentially provide a framework for further exploitation on clinical applications and drug delivery. 

Another promising commercial avenue for stimuli-responsive dendrimers is in the construction of the fourth architectural polymer class, which can lead to significant improvement in diagnosis, drug delivery, and therapy. Apart from drug delivery, dendrimers have shown great promise on delivery through I.V., ocular, nasal, oral, and transdermal routes. Promotion of commercialization of dendrimer technology may strengthen the benefits of these polymers in future applications. The application of dendrimers in targeted drug delivery; for example in CNS drug delivery, is also a promising area for future research. Thus, scientists should focus on the construction of more efficient and specific targets to ensure the creation of safer and effective dendrimers. However, extensive toxicological studies on noninvasive dendrimers, alternative drug delivery methods, and specific targeting still face many challenges that need to be addressed. The main goal of dendrimer use in industrial and biomedical fields is to facilitate safe and long-term application for diagnosis, drug delivery, and engineering, while at the same time minimizing the accumulation of adverse effects. Another promising future research area for dendrimer application is in their chemistry, which has been identified since the synthesis of the first dendrimers.

## Figures and Tables

**Table 1:** Examples of multi-stimuli-responsive dendrimer-based nanosystems as drug delivery for cancer treatment.

Polymer	Generation	Modification	Responsiveness	Payload	Ref.
PAMAM	G4	PEG	Redox and pH	Doxorubicin	[31]
PAMAM	G3.5	Heparin	Redox and pH	Letrozole	[32]
PAMAM	G4	PEG	Redox	Doxorubicin	[33]
PBAE	G4	2-(*N*,*N*-dimethylamino)ethyl acrylate	pH and temperature	Doxorubicin	[34]
PAMAM	G4	PEG-chitosan-folic acid	pH	pDNA	[35]

Note: Polyamidoamine: PAMAM. Poly(β-amino ester): PBAE.

**Table 2:** Examples of stimuli-responsive dendritic polymers.

	Stimuli	Responsive moiety	Ref.
Internal stimuli	Reduction	Au−S	[74]
		Disulfide	[75,76]
	pH	3,4,5,6-tetrahydrophthalic anhydride	[77]
		Hydrazine	[34,68,78]
	Temperature	Poly (NiPAM)	[79,80,81]
		Isobutyramide (IBAM)	[82]
		4-(isopropylamino)-4-oxobutanoic acid (IPAOBA) and 4-(diethylamino)-4-oxobutanoic acid (DEAOBA)	[83]
External stimuli	Light	Ortho-nitrobenzyl (ONB)	[51,84,85,86]
		4-(3-(trifluoromethyl)-3H-diazirin-3-yl) benzyl bromide	[87]
		Aso-nitrobenzyl and diazobenzene	[88]
	Ultrasound	Sonophoresis	[89,90,91,92,93,94]
	Magnetic field	Fe_3_O_4_@SiO_2_-NH_2_	[95]
	Enzyme	Gly-Phe-Leu-Gly (GFLG)	[96]
		Lysine	[97]
